# Pregnant Woman With Lower Abdominal Pain

**DOI:** 10.1016/j.acepjo.2025.100065

**Published:** 2025-02-15

**Authors:** Yi-Hsuan Hsieh, Siou-Ting Lee, Chen-Yu Wang

**Affiliations:** 1 Department of Obstetrics and Gynecology, Gangshan Branch of Zuoying Armed Forces General Hospital, Kaohsiung, Taiwan; 2 Department of Obstetrics and Gynecology, Tri-Service General Hospital, National Defense Medical Center, Taipei, Taiwan; 3 Department of Obstetrics and Gynecology, Taoyuan Armed Forces General Hospital, Taoyuan, Taiwan

**Keywords:** cervical insufficiency with prolapsed amniotic sac, cervical insufficiency, cerclage

## 1 Patient Presentation

A 39-year-old nulligravid woman at 21 weeks gestation presented to the emergency department with a 1-day history of lower abdominal pain. Physical examination revealed lower pelvic tenderness and occasional uterine contractions. There was no active bleeding or rupture of membranes. The pregnancy was conceived naturally, with prior prenatal checkups being normal. Abdominal ultrasonography showed a protruding amniotic sac, with the fetal legs and umbilical cord extending into the vagina.

## 2 Diagnosis: Cervical Insufficiency with Prolapsed Amniotic Sac

**Figure 1:**
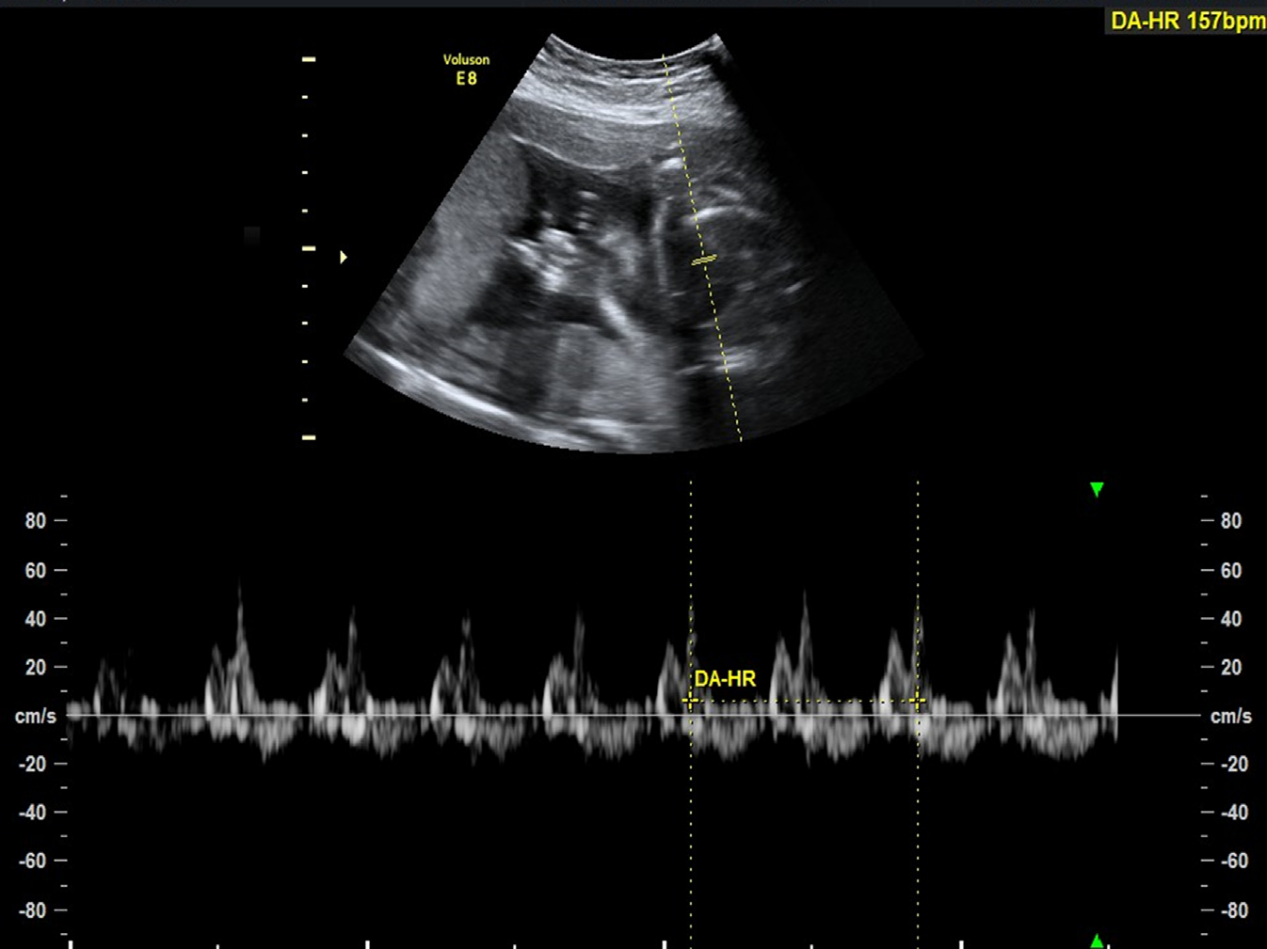
The fetal heart rate was 157 beats per minute.

**Figure 2:**
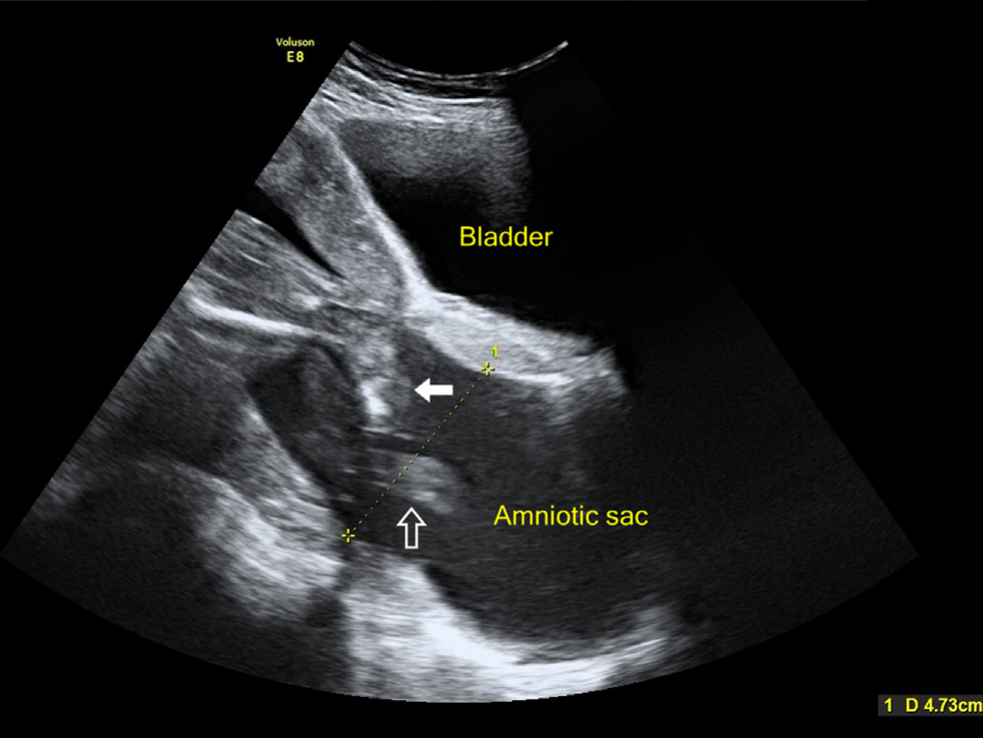
Abdominal ultrasonography revealed the amniotic sac bulging through the cervix, creating a classic “hourglass appearance,” with the fetal legs (Fig 2, arrow) and umbilical cord (Fig 2, hollow arrow) protruding. The cervix dilated to approximately 4.73 cm.

Abdominal ultrasonography revealed a fetal heart rate of 157 beats per minute (Fig 1). The amniotic sac was bulging through the cervix, creating a classic "hourglass appearance," with the fetal legs (Fig 2, arrow) and umbilical cord (Fig 2, hollow arrow) protruding. The cervix was dilated to approximately 4.73 cm (Fig 2), with an amniotic fluid index of 12.1 cm and no signs of membrane rupture. These findings were consistent with cervical insufficiency, a primary cause of amniotic sac prolapse.^1^ An emergent cerclage was performed uneventfully.^2^^,^^3^

Emergency physicians and obstetricians should prioritize abdominal ultrasonography over pelvic examination in pregnant women at or beyond 20 weeks of gestation. Unlike the common emergency department practice of performing a pelvic examination first, ultrasonography can identify critical conditions such as placenta previa or a protruding sac, where a pelvic examination could risk massive hemorrhage,^4^ sac rupture, preterm labor, or infection. Abdominal ultrasonography offers more valuable information to guide appropriate management.

## Conflict of Interest

All authors have affirmed they have no conflicts of interest to declare.
